# Inherited human group IVA cytosolic phospholipase A_2_ deficiency abolishes platelet, endothelial, and leucocyte eicosanoid generation

**DOI:** 10.1096/fj.15-275065

**Published:** 2016-10-17

**Authors:** Nicholas S. Kirkby, Daniel M. Reed, Matthew L. Edin, Francesca Rauzi, Stefania Mataragka, Ivana Vojnovic, David Bishop-Bailey, Ginger L. Milne, Hilary Longhurst, Darryl C. Zeldin, Jane A. Mitchell, Timothy D. Warner

**Affiliations:** * National Heart and Lung Institute, Imperial College London, London, United Kingdom;; † William Harvey Research Institute, Queen Mary University of London, London, United Kingdom;; ‡ National Institutes of Health, National Institute of Environmental Health Sciences, Research Triangle Park, North Carolina, USA;; § Department of Comparative Biomedical Sciences, Royal Veterinary College, London, United Kingdom;; ¶ Department of Pharmacology and Department of Medicine, Vanderbilt University, Nashville, Tennessee, USA; and; ‖ Immunology Department, Barts Health and the London National Health Service Trust, London, United Kingdom

**Keywords:** cardiovascular, thromboxane A_2_, prostacyclin, inflammation

## Abstract

Eicosanoids are important vascular regulators, but the phospholipase A_2_ (PLA_2_) isoforms supporting their production within the cardiovascular system are not fully understood. To address this, we have studied platelets, endothelial cells, and leukocytes from 2 siblings with a homozygous loss-of-function mutation in group IVA cytosolic phospholipase A_2_ (cPLA_2_α). Chromatography/mass spectrometry was used to determine levels of a broad range of eicosanoids produced by isolated vascular cells, and in plasma and urine. Eicosanoid release data were paired with studies of cellular function. Absence of cPLA_2_α almost abolished eicosanoid synthesis in platelets (*e.g.*, thromboxane A_2_, control 20.5 ± 1.4 ng/ml *vs.* patient 0.1 ng/ml) and leukocytes [*e.g.*, prostaglandin E_2_ (PGE_2_), control 21.9 ± 7.4 ng/ml *vs.* patient 1.9 ng/ml], and this was associated with impaired platelet activation and enhanced inflammatory responses. cPLA_2_α-deficient endothelial cells showed reduced, but not absent, formation of prostaglandin I_2_ (prostacyclin; control 956 ± 422 pg/ml *vs.* patient 196 pg/ml) and were primed for inflammation. In the urine, prostaglandin metabolites were selectively influenced by cPLA_2_α deficiency. For example, prostacyclin metabolites were strongly reduced (18.4% of control) in patients lacking cPLA_2_α, whereas PGE_2_ metabolites (77.8% of control) were similar to healthy volunteer levels. These studies constitute a definitive account, demonstrating the fundamental role of cPLA_2_α to eicosanoid formation and cellular responses within the human circulation.—Kirkby, N. S., Reed, D. M., Edin, M. L., Rauzi, F., Mataragka, S., Vojnovic, I., Bishop-Bailey, D., Milne, G. L., Longhurst, H., Zeldin, D. C., Mitchell, J. A., Warner, T. D. Inherited human group IVA cytosolic phospholipase A_2_ deficiency abolishes platelet, endothelial, and leucocyte eicosanoid generation.

In the cardiovascular system, eicosanoids have well-characterized roles in both normal function and a range of disease states (1, 2). For example, thromboxane A_2_ (TXA_2_), generated by platelets, drives thrombotic responses to particular stimuli (*e.g.*, collagen) and contributes to atherogenesis, whereas prostaglandin I_2_ (prostacyclin), generated by endothelial cells, causes vasodilatation, inhibits platelet activation, and suppresses vascular inflammation. In leukocytes, eicosanoid formation [predominantly prostaglandin E_2_ (PGE_2_)] is induced by proinflammatory stimuli such as LPS that up-regulate cyclooxygenase (COX)-2 and other biosynthetic pathways (3) and so modulate the inflammatory response. In each case, although specific eicosanoid pathways such as TXA_2_, PGE_2_, and prostacyclin are well characterized, platelets, endothelial cells, and leukocytes synthesize substantial amounts of other arachidonic acid–derived mediators, the effect of which in combination remains poorly understood.

The arachidonic acid required for eicosanoid production is released from the sn-2 position of membrane glycerophospholipids by the actions of phospholipase A_2_ (PLA_2_) enzymes. As reviewed (4, 5), >30 PLA_2_ enzymes have been identified and currently classified into 6 broad families: secreted phospholipase A_2_ (sPLA_2_), Ca^2+^-dependent cytosolic phospholipase A_2_ (cPLA_2_), calcium-independent phospholipase A_2_ (iPLA_2_), platelet-activating factor acetylhydrolases, lysosomal PLA_2_, and adipose-specific phospholipase. Of the known isoforms, group IVA cPLA_2_ (also referred to as cPLA_2_α), encoded by the PLA2G4A gene, is the most studied and has been characterized as a cytosolic enzyme, which upon Ca^2+^-dependent activation cleaves arachidonate-containing phospholipids to generate free intracellular arachidonic acid. This arachidonic acid is then used as a substrate by enzymes that synthesize the eicosanoid mediators, including COXs that produce prostanoids such as TXA_2_ and prostacyclin, lipoxygenases (LOXs) that generate hydroxyeicosatetraenoic acids (*e.g.*, 12-HETE), and cytochrome P450 enzymes that generate epoxyeicosatrienoic acids (EETs) and HETEs (4, 5). cPLA_2_α is widely expressed through the vasculature, in platelets, and in most types of blood leukocytes. Nonetheless, vascular and blood cells are known to express other PLA_2_ enzymes, such as sPLA_2_ enzymes including group II (platelets) and group V (endothelium) isoforms as well as iPLA_2_ isoforms, which could also liberate arachidonic acid. For example, exogenous sPLA_2_ has been demonstrated to activate platelets (6, 7) and endothelial cells (8). A role for endogenous sPLA_2_ and iPLA_2_ enzymes in eicosanoid generation by agonist-stimulated platelets (7, 9, 10), endothelial cells (11, 12), and leukocytes (13, 14) has also been described by several groups, calling into question the relative role of PLA_2_ isoforms in eicosanoid generation and vascular protection. Indeed, the recent failure of the sPLA_2_ inhibitor varespladib for the prevention of cardiovascular events in patients with acute coronary syndromes underlines our inadequate knowledge of the role of PLA_2_ enzymes in vascular health and disease (15).

The key role of cPLA_2_α in the generation of eicosanoid mediators is supported by data from cPLA_2_α-knockout mice (9) and pharmacologic inhibitors (16, 17). Furthermore, we have recently reported 2 siblings with a homozygous mutation of the PLA2G4A gene that leads to a complete absence of cPLA_2_α activity (18). Our work (18) and similar work from 2 other groups (19, 20) using tissue from patients with a heterozygous mutation of the PLA2G4A gene has shown that cPLA_2_α regulates production of particular eicosanoids in platelets and in the urine. However, the relative role of cPLA_2_α in endothelial cell and leukocyte eicosanoid function, as well as more broadly in platelets, has not thus far been addressed. By performing such studies, we have now definitively defined and compared the contribution of cPLA_2_α with eicosanoid formation and inflammatory responses in leukocytes, platelets, and in endothelial cells. Our data show, for the first time, how loss of this fundamental enzyme system regulates phenotypes and inflammatory responses of these cardiovascular cells and associated urinary markers relevant to vascular disease.

## MATERIALS AND METHODS

### Blood collection and ethics

Blood was collected by venipuncture from healthy volunteers and from 2 patients (brother, patient B; sister, patient S) bearing a homozygous mutation in the PLA2G4A gene, which disrupts the active site of cPLA_2_α (18). All experiments were subject to written informed consent, local ethical approval (healthy volunteer samples for platelet/leukocyte studies; St. Thomas’s Hospital Research Ethics Committee, reference 07/Q0702/24: endothelial cell studies; Royal Brompton and Harfield Hospital Research Ethics Committee, reference 08/H0708/69: patient samples; South East National Health Service Research Ethics Committee), and in accordance with Declaration of Helsinki principles.

### Whole-blood stimulation

Heparin-anticoagulated whole blood was incubated with vehicle (PBS), Horm collagen (Nycomed, St. Peter, Austria), thrombin receptor-activating peptide (TRAP)-6 amide (Bachem, Heidelberg, Germany), Ca^2+^ ionophore, A23187 (Sigma-Aldrich, Poole, United Kingdom), for 30 min, or with LPS (Sigma-Aldrich), triacylated lipoprotein CSK4 (Pam3CSK4; InvivoGen, Toulouse, France), bisacylated lipoprotein CGDPKHPKSF (FSL-1; InvivoGen), polyinosinic:polycytidylic acid [poly(I:C); Sigma-Aldrich], or IL-1β (Invitrogen, Life Technologies, Paisley, United Kingdom) for 18 h in the presence or absence of diclofenac (10 μM; Sigma-Aldrich). Levels of (C-X-C motif) ligand-8 (CXCL8; R&D Systems, Abingdon, United Kingdom), PGE_2_ (Cisbio, Saclay, France), or TXB_2_ (Cayman Chemical, Cambridge Bioscience, Cambridge, United Kingdom) were measured by immunoassay or total eicosanoids by gas chromatography–tandem mass spectrometry (see below) in the conditioned plasma.

### Eicosanoid analysis

Basal and conditioned plasma was subject to eicosanomic analysis as previously described (21). Urinary prostanoid levels were determined by gas chromatography–tandem mass spectrometry as previously described (22, 23).

### Light transmission aggregometry and ATP release

Platelet-rich plasma was preincubated with the COX inhibitor aspirin (30 μM; Sigma-Aldrich), the cPLA_2_ inhibitor pyrrophenone (40 μM; Cayman Chemical, Cambridge Bioscience), or vehicle for 30 min at 37°C. Aggregation and ATP secretion responses to collagen (0.3–3 μg/ml), ADP (5 μM; Chrono-log; Labmedics, Abingdon, United Kingdom), U46619 (10 μM; Enzo Life Sciences, Exeter, United Kingdom), or arachidonic acid (1 mM; Sigma-Aldrich) were measured using a Chrono-log 560CA Lumi-Aggregometer (Chrono-log Corp., Havertown, PA, USA).

### Platelet adhesion under flow

Whole blood was preincubated with aspirin (100 μM), pyrrophenone (40 μM), or vehicle before labeling of cells with mepacrine (10 μM; Sigma-Aldrich) for a further 30 min. This was then drawn through a slide chamber (Ibidi, Munich, Germany) coated with collagen (100 μg/ml) by a syringe pump to achieve a shear rate of 1000 s^−1^.

### Endothelial cells

Blood outgrowth endothelial cells were grown out from progenitors in human blood as previously described (24–27). Once colonies emerged (between d 4 and 20), cells were expanded and maintained in Lonza EGM-2 medium (Lonza, Slough, United Kingdom) plus 10% fetal bovine serum, and experiments were performed between passages 2 and 8.

### Endothelial cell immunocytochemistry

Endothelial cells were stained using anti-CD31 (platelet endothelial cell adhesion molecule-1)–Alexa Fluor 488 (BioLegend, London, United Kingdom) or anti-vascular endothelial-cadherin (Santa Cruz Biotechnology, Dallas, TX, USA) and imaged using a Cellomics VTi HCS Arrayscanner (Thermo Fisher Scientific, Hemel Hempstead, United Kingdom).

### Endothelial cell eicosanoid and cytokine production

Cells were plated on 48- or 96-well plates. For eicosanoid measurements, endothelial cells were primed with IL-1β (1 ng/ml) to up-regulate COX pathways, as described previously (28), before being treated for 30 min with A23187 or thrombin to activate PLA_2_ or arachidonic acid to supply eicosanoid substrate directly. For inflammation studies, endothelial cells were treated with vehicle (Lonza EGM-2) or TLR ligands: heat-killed *Listeria monocytogenes* (10^7^ cells/ml), Pam3CSK4 (1 μg/ml), FSL-1 (1 μg/ml), poly(I:C) (10 μg/ml), LPS (1 μg/ml), *Staphylococcus aureus*–derived flagellin (FLA; 100 ng/ml), imiquimod (1 μg/ml), single-stranded RNA oligonucleotide-40 (1 μg/ml), and oligodeoxynucleotide-2006 (5 μM). After 24 h, media were collected for measurement of CXCL8 release by ELISA (R&D Systems).

### Statistics and data analysis

Data are expressed as means ± se. Statistical analysis was performed by 1- or 2-way ANOVA or by unpaired Student’s *t* test using GraphPad Prism 6 software (GraphPad Software, La Jolla, CA, USA). Patient eicosanomics data (*n* = 1–2) were interpreted qualitatively without statistical testing.

## RESULTS

### Role of cPLA_2_α in eicosanoid formation in platelets

**Figure 1.**
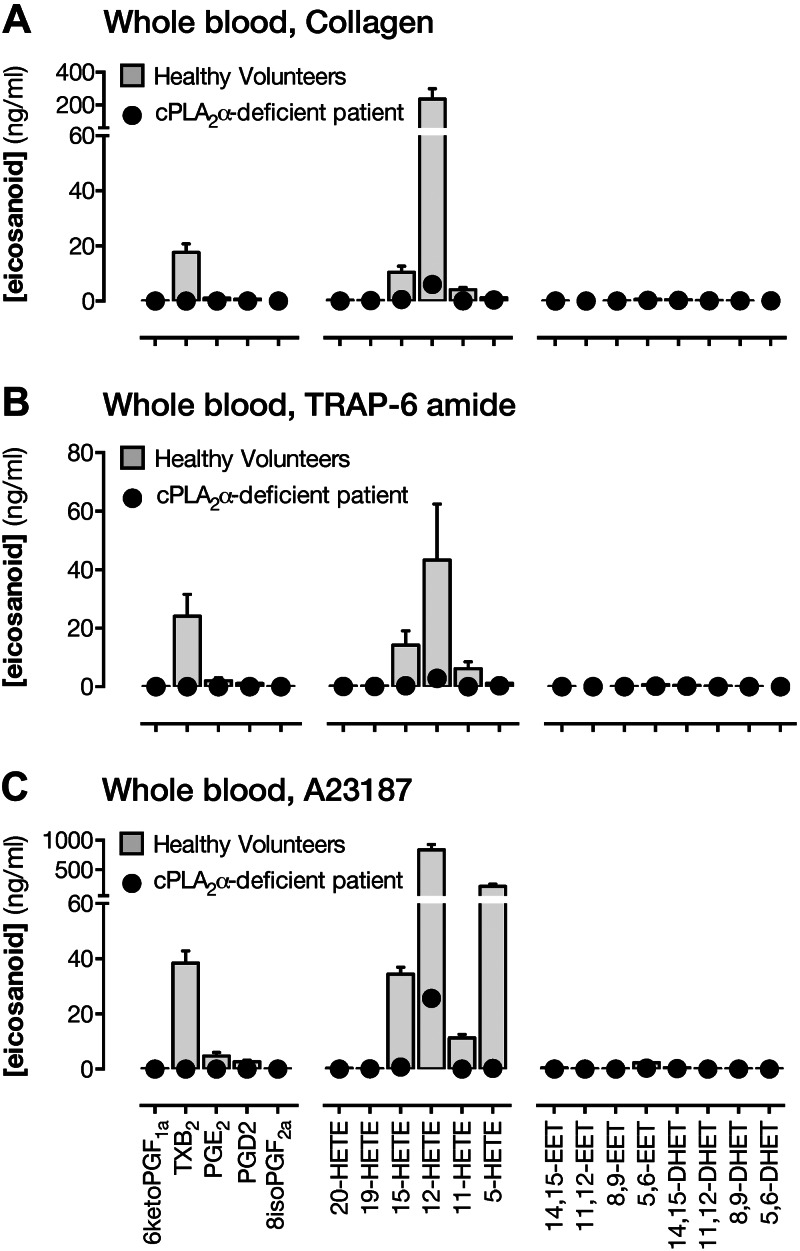
Contribution of cPLA_2_α to eicosanoid synthesis in whole blood. Eicosanoid levels in whole blood from healthy volunteers or from a patient lacking cPLA_2_α stimulated with collagen (30 μg/ml) (*A*), TRAP-6 amide (30 μM) (*B*), or A23187 Ca^2+^ ionophore (50 μM) (*C*). Levels are expressed as increase over levels in vehicle-treated blood. *n* = 3–6 (healthy volunteers); *n* = 1 (patient S).

Incubation of blood with collagen (**Fig. 1*A***) or TRAP-6 (Fig. 1*B*) to specifically activate platelets increased levels of TXB_2_ (the stable breakdown product of TXA_2_) and 12-HETE, in particular. There were also increases in PGE_2_, prostaglandin D_2_ (PGD_2_), 11-HETE, and 15-HETE. 12-HETE levels were somewhat lower in TRAP-6–stimulated blood as compared with collagen-stimulated blood. In blood treated with the Ca^2+^ ionophore, A23187, to cause acute receptor-independent activation of platelets and leukocytes, a broadly similar pattern of eicosanoid formation was observed (Fig. 1*C*) with a marked production of 12-HETE, followed by TXB_2_, 15-HETE, and 11-HETE. There were also greatly increased levels of 5-HETE, representing acute activation of leukocytes, and a more modest production of 5,6-EET. In each case, eicosanoid production to these stimuli was almost absent in blood from cPLA_2_α-deficient patients (Fig. 1 and Supplemental Tables S1 and S2). Normal eicosanoid formation was observed in the presence of exogenous arachidonic acid in both healthy volunteer and cPLA_2_α-deficient patient blood. In isolated platelets (platelet-rich plasma), TXB_2_ formation induced by ADP, collagen, or the TXA_2_-mimetic U46619, but not exogenous arachidonic acid, was abolished by cPLA_2_α deficiency (Supplemental Fig. S1).

### Role of cPLA_2_α in platelet aggregation, secretion, and adhesion responses

**Figure 2.**
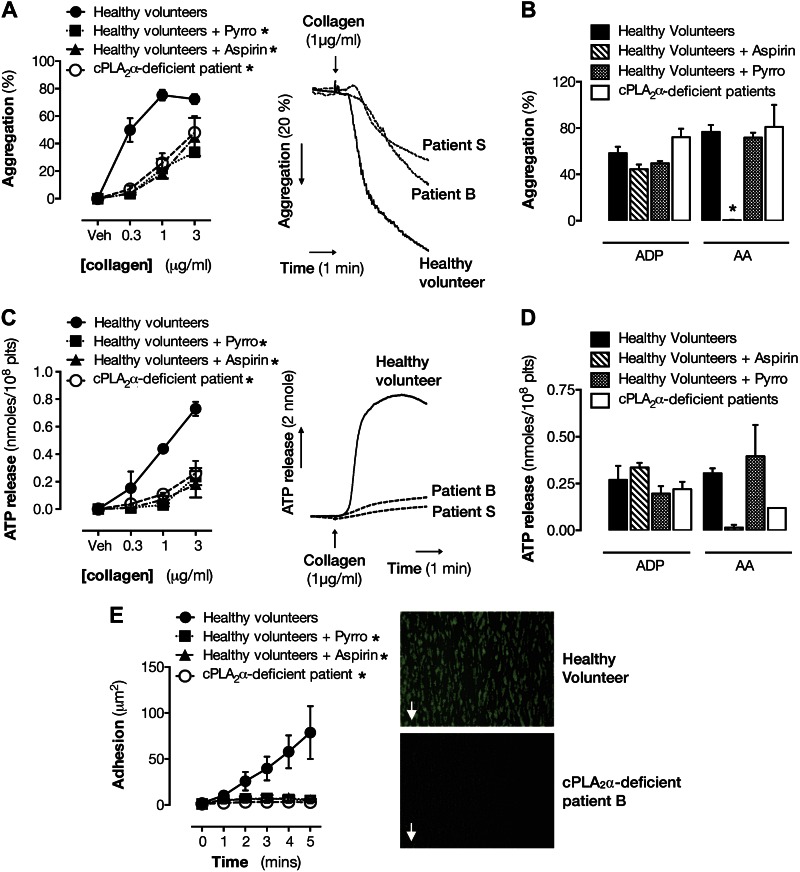
Effect of cPLA_2_α deficiency on platelet aggregation, secretion, and adhesion responses. Effect of cPLA_2_α deficiency, cPLA_2_α inhibition, and aspirin on platelet aggregation to collagen (0.1–3 μg/ml) (*A*), ADP (5 μM) (*B*), and arachidonic acid (AA; 1 mM) is shown. Pyrro, pyrrophenone. ATP secretion to collagen (0.1–3 μg/ml) (*C*), ADP (5 μM) (*D*), and arachidonic acid (1 mM). *E*) Platelet adhesion to collagen under flow (1000 s^−1^). *n* = 2–4 (healthy volunteers); *n* = 2 (patient B and patient S). *P < 0.05 by 2-way ANOVA with Dunnett’s posttest.

Absence of cPLA_2_α or cPLA_2_ inhibition by pyrrophenone produced a marked reduction in collagen-induced aggregation similar to that produced by aspirin (**Fig. 2*A***) but had little effect upon responses to ADP or exogenous arachidonic acid (Fig. 2*B*). ATP release induced by collagen (Fig. 2*C*), but not that induced by ADP or arachidonic acid (Fig. 2*D*), was strongly blunted by loss of functional cPLA_2_α or aspirin treatment, and under flow conditions, platelet adhesion to collagen was almost abolished by cPLA_2_α inhibition/deficiency (Fig. 2*E*).

### Role of cPLA_2_α in eicosanoid formation in endothelial cells

**Figure 3.**
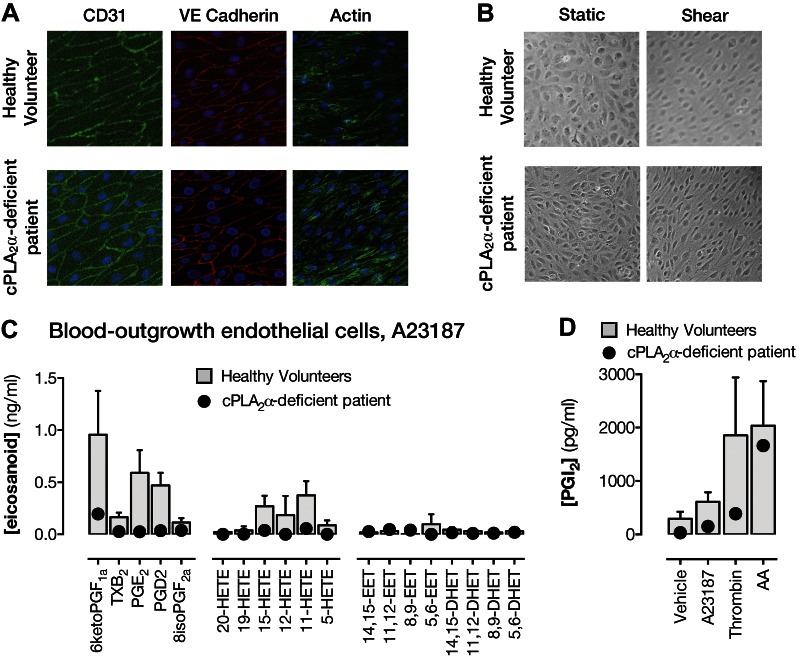
Phenotyping of and eicosanoid synthesis by endothelial cells grown out of blood progenitors from healthy volunteers and from a cPLA_2_α-deficient patient. *A*) Endothelial-specific marker expression of CD31 (green) and VE cadherin (red) and actin staining (green). *B*) Morphologic response to shear stress after 3 d. *C*) Eicosanoid release in IL-1β (1 ng/ml)-primed endothelial cells stimulated with A23187 (50 μM). *D*) Prostacyclin release from IL-1β–primed endothelial cells stimulated for 30 min with A23187 (50 μM), thrombin (1 U/ml), or arachidonic acid (AA; 50 μM). Data are from at least 3 wells per condition. *n* = 3–6 (healthy volunteers); *n* = 1 (patient S).

Endothelial cells from healthy volunteers or derived from cPLA_2_α-deficient patients emerged in culture after 4–20 d, grew with typical cobblestone morphology, expressed the endothelial cell markers CD31 and VE cadherin (**Fig. 3*A***), and aligned when cultured under directional shear stress (29) (Fig. 3*B*). In the presence of A23187, endothelial cells from healthy volunteers released predominately prostacyclin (measured as the stable breakdown product 6-keto-PGF_1_α) followed by PGE_2_, PGD_2_, 11-HETE, and 15-HETE. In each case, eicosanoid release was lower but not abolished in endothelial cells derived from cPLA_2_α-deficient patients (Fig. 3*C*) (*e.g.*, prostacyclin release from cPLA_2_α-deficient endothelial cells was reduced by ∼80%). Similarly, the cPLA_2_ inhibitor, pyrrophenone, produced a concentration-dependent inhibition of prostacyclin release from endothelial cells grown from healthy donors (Supplemental Fig. S2), with a maximal effect of ∼80%. Prostacyclin was also released from endothelial cells of healthy volunteers when stimulated with the receptor-dependent activator, thrombin (Fig. 3*D*). As described for A23187-stimulated release above, thrombin-stimulated prostacyclin release was reduced but not abolished in cPLA_2_α-deficient patient endothelial cells. Endothelial cells of both genotypes responded strongly to exogenous arachidonic acid (Fig. 3*D*).

### Role of cPLA_2_α in eicosanoid formation by leukocytes

**Figure 4.**
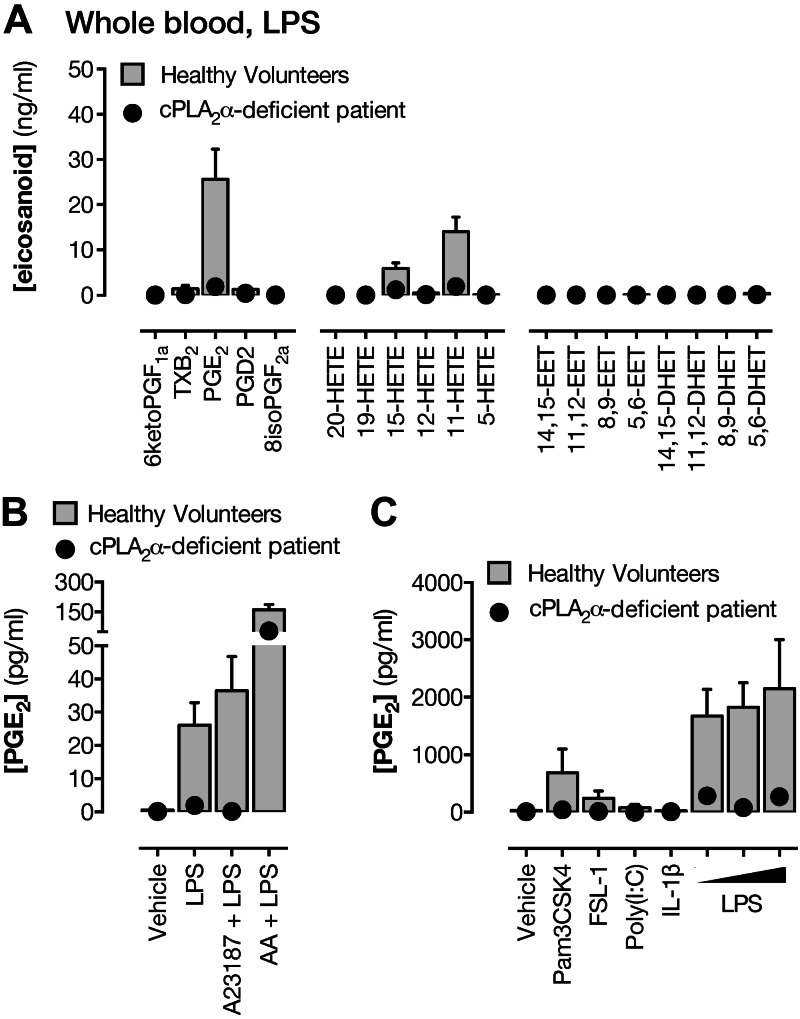
Contribution of cPLA_2_α to eicosanoid synthesis in leukocytes. *A*) Eicosanoid levels in whole blood from healthy volunteers or from a patient lacking cPLA_2_α treated with LPS (10 μg/ml) for 18 h. PGE_2_ formation in whole blood treated with LPS (10 μg/ml) for 18 h followed by addition of A23187 (50 μM) or arachidonic acid (AA; 1 mM) for 30 min (*B*) or treated with agonists to TLR2/1 (Pam3CSK4; 1 μg/ml), TLR2/6 (FSL-1; 1 μg/ml), TLR3 [poly(I:C); 10 μg/ml], IL-1 receptor (IL-1β; 1 ng/ml), or TLR4 (LPS; 5–20 μg/ml) (*C*). *n* = 3–6 (healthy volunteers); *n* = 1 (patient S).

When whole blood was stimulated (18 h) with the TLR4 agonist, LPS, to activate leukocytes and inducible biosynthetic pathways, the major eicosanoids produced were 12- and 15-HETE and PGE_2_, and a smaller amount of 11-HETE (**Fig. 4*A*** and Supplemental Table S3). In cPLA_2_α-deficient patient blood, LPS-induced production of PGE_2_ and 15-HETE was greatly reduced, whereas the production of 12-HETE was little affected. Overall, productions were restored by acute addition of arachidonic acid (Fig. 4*B* and Supplemental Table S3). Pam3CSK4 (TLR2/1) and FSL-1 (TLR2/6), which activate pattern recognition receptors associated with gram-positive bacteria, as with LPS, activated whole blood to release PGE_2_, an effect that was abolished by cPLA_2_α deficiency (Fig. 4*C*). Neither Poly(I:C), which stimulates the viral pathogen recognition receptor TLR3, nor IL-1β, which works independently of pattern recognition receptors, stimulated PGE_2_ release from whole blood.

### Role of cPLA_2_α in inflammatory responses in endothelial cells and blood leukocytes

**Figure 5.**
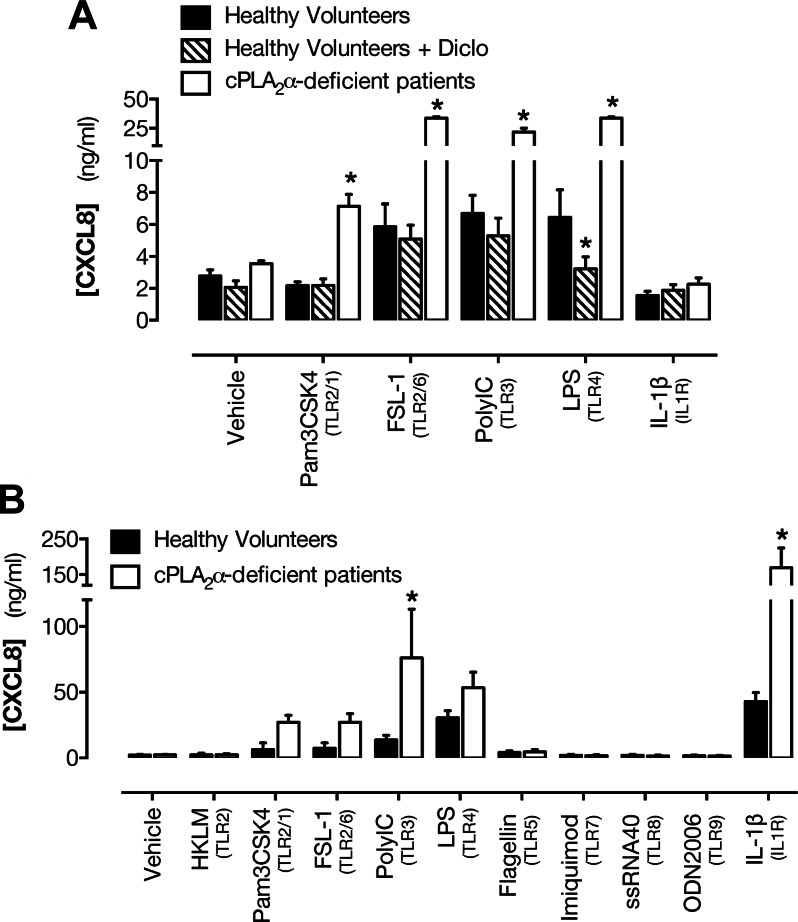
Effect of cPLA_2_α deficiency on blood leukocyte and endothelial cell inflammatory responses. *A*) CXCL8 release in whole blood from healthy volunteers with or without pretreatment with the COX inhibitor diclofenac (Diclo; 10 μM) or a cPLA_2_α-deficient patient in response to agonists to TLR2/1 (Pam3CSK4; 1 μg/ml), TLR2/6 (FSL-1; 1 μg/ml), TLR3 [poly(I:C); 10 μg/ml], TLR4 (LPS; 1 μg/ml), or IL-1 receptor (IL-1β; 1 ng/ml). *B*) CXCL8 release by endothelial cells to agonists of TLR2 [heat-killed *L. monocytogenes* (HKLM); 10^7^ cells/ml], TLR2/1 (Pam3CSK4; 1 μg/ml), TLR2/6 (FSL-1; 1 μg/ml), TLR3 [poly(I:C); 10 μg/ml], TLR4 (LPS; 10 μg/ml), TLR5 (FLA; 100 ng/ml), TLR7 (imiquimod; 1 μg/ml), TLR8 [single-stranded RNA oligonucleotide-40 (ssRNA40); 1 μg/ml], TLR9 [oligodeoxynucleotide-2006 (ODN2006); 5 μM], or IL-1 receptor (IL-1β; 1 ng/ml). *n* = 3–6 (healthy volunteers; 2 determinations each); *n* = 1 (patient S; 3 determinations each). *P < 0.05 by 2-way ANOVA with Dunnett’s posttest.

Whole blood from healthy volunteers treated with FSL-1, Poly(I:C), or LPS, but not with IL-1β, released the inflammatory chemokine CXCL8 (**Fig. 5*A***). Blood from a cPLA_2_α-deficient patient exhibited more than 5-fold greater responses to all agents except IL-1β as compared with matched controls (Fig. 5*A*). Treatment of blood from healthy volunteers with the COX inhibitor diclofenac suppressed the CXCL8 response to LPS but did not modify CXCL8 release stimulated by other tested agents (Fig. 5*A*).

Endothelial cells from healthy donors also released CXCL8 when stimulated with pathogen-associated molecular patterns (PAMPs) directed at TLR2, 3, or 4, or with IL-1β. Again, as with leukocytes in whole blood, endothelial cells from a cPLA_2_α-deficient patient released elevated levels of CXCL8 when stimulated with inflammatory agents (Fig. 5*B*). Endothelial cells from either type of donor did not respond to ligands for TLR5, TLR7, or TLR8 (Fig. 5*B*).

### Involvement of cPLA_2_α in plasma and urinary eicosanoid levels

**Figure 6.**
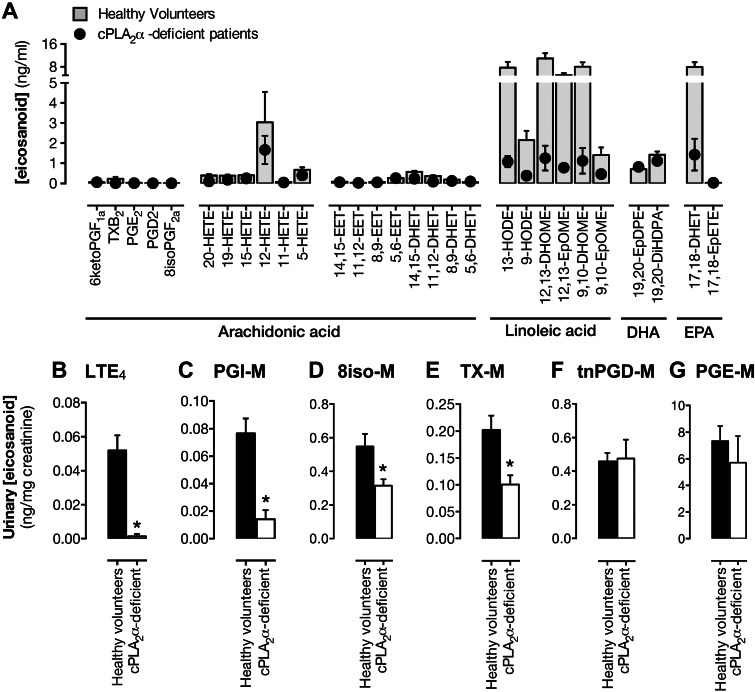
Contribution of cPLA_2_α to basal plasma and urinary eicosanoid levels. *A*) Basal eicosanoid levels in plasma from healthy volunteers (circles) or from a patient lacking cPLA_2_α. *n* = 8 (healthy volunteers; 2 determinations each); *n* = 2 (patient B and patient S; 2 determinations each). Urinary levels of LTE_4_ (*B*) and metabolites of prostacyclin (PGI-M) (*C*), 8-isoprostane (8iso-M) (*D*), TXA_2_ (TX-M) (*E*), PGD_2_ [tetranor (tn)PGD-M] (*F*), and PGE_2_ (PGE-M) (*G*) in healthy volunteers (filled columns) or from patients lacking cPLA_2_α (unfilled columns). *n* = 7 (healthy volunteers; 2 determinations each); *n* = 2 (patient B and patient S; 4 determinations each).

Plasma from healthy volunteers contained primarily metabolites of linoleic acid, eicosapentaenoic acid (EPA), and docosahexaenoic acid (DHA). Patients lacking cPLA_2_α had reduced levels of these mediators compared with plasma from healthy volunteers (**Fig. 6*A***). Basal plasma also contained substantial levels of 12-HETE, and this remained in cPLA_2_α-deficient patient plasma.

Absence of cPLA_2_α was associated with strong reductions in the levels of leukotriene E4 (LTE_4_) and prostacyclin metabolites (Fig. 6*B*, *C*), whereas substantial levels of PGD_2_, PGE_2_, and 8-isoprostane metabolites remained (Fig. 6*D*–*G*). Levels of the urinary metabolite of thromboxane A_2_ (TX-M) were 50% lower in cPLA_2_α-deficient patients as compared with healthy volunteers (0.202 ± 0.010 ng/mg creatinine *vs.* 0.101 ± 0.017 ng/mg creatinine; *P* < 0.01; Fig. 6*E*). In addition, substantial levels of PGD_2_, PGE_2_, and 8-isoprostane metabolites remained in urine samples from cPLA_2_α-deficient patients.

## DISCUSSION

Here, we have examined the contribution of cPLA_2_α to eicosanoid formation, and thrombotic and inflammatory responses in platelets, blood leukocytes, and endothelial cells from 2 individuals with a unique genetic inactivation of this enzyme. Although we (18) and others (19, 20) have published reports of individuals lacking functional cPLA_2_α, including limited analysis of platelet responses, this is the first time a full and systematic eicosanoid analysis has been undertaken on samples from these patients and considered in the context of the circulatory system in health and inflammation. These data demonstrate an absolute requirement for cPLA_2_α in eicosanoid synthesis in the vascular compartment with a consequent loss of platelet activation pathways, reduced antithrombotic prostacyclin, and increased inflammatory sensitivity of both endothelial cells and leukocytes.

### Platelets

The PLA_2_ system in platelets is among the most clearly defined in cardiovascular cell types. We and others have previously performed limited phenotyping of platelets from cPLA_2_α-deficient individuals and found a requirement for this enzyme in TXA_2_ formation and collagen-induced platelet aggregation, which is TXA_2_ dependent. However, in addition to cPLA_2_α, platelets also express sPLA_2,_ which others suggest contributes to eicosanoid formation in platelets (9, 10, 30). Here, for the first time, we have performed a full eicosanomic analysis of samples from cPLA_2_α-deficient individuals to consider the role of this enzyme in synthesis of a range of functional distinct arachidonic acid–derived mediators. Stimulation of whole blood with the platelet activators collagen or TRAP-6 resulted in greatly increased synthesis of TXA_2_, in addition to PGE_2_, PGD_2_, and 11-, 12-, and 15-HETE, mediators primarily produced by COX and LOX pathways. 12-HETE levels were somewhat lower in TRAP-6–stimulated blood as compared to collagen-stimulated blood, consistent with reports that 12-LOX activation is linked to the platelet collagen receptor, glycoprotein VI (7). Stimulation of blood with the receptor-independent activator Ca^2+^ ionophore A23187 produced a similar platelet eicosanoid fingerprint, but unlike collagen and TRAP-6, increased levels of 5-HETE, reflecting acute activation of leukocytes. In each case, eicosanoid production was cPLA_2_α dependent because it was lost in cPLA_2_α-deficient patient blood but reversed by the addition of exogenous arachidonic acid, demonstrating its dependence on loss of endogenous substrate release. In agreement, isolated cPLA_2_α-deficient platelets stimulated with a range of agonists (collagen, ADP, and U46619), but not exogenous arachidonic acid, exhibited a complete loss of TXA_2_ synthesis, in contrast to reports that ADP-induced release is not altered in cPLA_2_α-knockout mouse platelets (9). These data illustrate the requirement for cPLA_2_α in the full range of eicosanoids synthesized by platelets and that this is independent of the stimulus used (9, 10).

We next set out to establish the contribution of cPLA_2_α-derived eicosanoids to functional platelet aggregation responses. Although it is well known that the platelet COX product TXA_2_ is a powerful proaggregatory agent, this response may be modified by other eicosanoid mediators synthesized in parallel. Indeed, PGE_2_ (31), 12-HETE (32–34), 15-HETE (35), and 5,6-EET (36) increase platelet activation, whereas PGD_2_ (37) and higher levels of PGE_2_ may limit platelet activation (31), meaning the net contribution of cPLA_2_α-derived eicosanoids is unclear. Our studies using traditional light transmission lumi-aggregometry and 96-well plate aggregometry demonstrated that inhibition or absence of cPLA_2_α produced a marked reduction in collagen-induced platelet aggregation and dense granule (ATP) secretion, in agreement with what we (18) and others (9, 19, 20) have previously reported. These defects were rescued by exogenous arachidonic acid, demonstrating that they were specifically associated with loss of endogenous substrate release. Similarly, platelet adhesion to a collagen-coated surface in flowing blood was abolished by cPLA_2_ inhibition and absent in blood from cPLA_2_α-deficient patients. These data are in agreement with reports of the importance of cPLA_2_α and TXA_2_ generation in platelet adhesion (38). In each of these functional assays, the reduction observed was similar to that produced by the COX inhibitor aspirin, suggesting that regulation of collagen-induced platelet responses by cPLA_2_α is due to products of platelet COX-1, probably TXA_2_. Overall, these data show that cPLA_2_α is absolutely required for formation of eicosanoid mediators in platelets and that despite the synthesis of several eicosanoid families, the contribution of cPLA_2_α to platelet aggregation, secretion, and adhesion responses can be entirely accounted for by generation of COX products. This reduction in platelet function is consistent with an increased tendency to bruising noted in the clinical care of these patients.

### Endothelium

Through eicosanoid release, endothelial cells are key to health and disease of the circulation. Here, we have made use of endothelial cells isolated from blood progenitors providing the first opportunity to study genetic deficiency of cPLA_2_α in human endothelium. Endothelial cells from a cPLA_2_α-deficient patient expressed the normal endothelial cell markers CD31 and vascular endothelial-cadherin, had a cobblestone morphology, and when cultured under conditions of chronic (3 d) laminar shear stress (29), aligned with the direction of shear, demonstrating their endothelial phenotype. When we examined eicosanoid production by endothelial cells, A23187 stimulation increased production of several eicosanoid mediators, the most abundant of which was prostacyclin, with lower levels of PGE_2_, PGD_2_, and 11-, 12-, and 15-HETE. These were predominantly driven by cPLA_2_α because they were strongly reduced in cPLA_2_α-deficient endothelial cells. This was further confirmed by the ability of a selective cPLA_2_ inhibitor to prevent the majority of A23187-stimulated prostacyclin production by endothelial cells and was specific because cPLA_2_α-deficient endothelial cells responded normally to exogenous arachidonic acid. However, cPLA_2_α-deficient endothelial cells stimulated with either A23187 or thrombin continued to produce some prostacyclin, probably reflecting contribution of other PLA_2_ isoforms [*e.g.*, group VIA iPLA_2_ (also referred to as iPLA_2_β)] to eicosanoid generation in endothelium (11, 12).

### Leukocytes and inflammation

In parallel with platelet and endothelial cell studies, we examined the effect of addition of inflammatory stimuli (*e.g.*, LPS) to whole blood to investigate the role of cPLA_2_α in blood leukocyte responses, an approach frequently applied in the eicosanoid field (3, 39). When whole blood was stimulated with A23187, in addition to platelet-derived mediators, 5-HETE was detected, which is associated with 5-LOX present in monocytes and neutrophils (40). When blood was stimulated with LPS to specifically activate leukocytes and inducible biosynthetic pathways such as COX-2, a more characteristic inflammatory eicosanoid profile was produced with PGE_2_, 12- and 15-HETE being the most abundant products. In each case, eicosanoid synthesis was cPLA_2_α mediated. In cPLA_2_α-deficient patient blood, LPS-induced production of PGE_2_ and 15-HETE was greatly reduced, and overall, productions were restored by acute addition of arachidonic acid, confirming that this defect is due to loss of free endogenous arachidonic acid. In contrast, in LPS-stimulated blood, the production of 12-HETE was little affected by cPLA_2_α deficiency, suggesting that other PLA_2_ isoforms specifically couple to 12-HETE synthesis in blood leukocytes. By its actions on TLR4, LPS mimics the effects of gram-negative bacteria. However, other pathogens activate immune and inflammatory responses in tissues through different pattern recognition receptor signaling pathways, each of which could theoretically drive eicosanoid production by different PLA_2_ isoforms. To address this, we studied the effect of a full range of PAMPs that mimic gram-positive, as well as gram-negative, bacteria or viruses. Thus, Pam3CSK4 (TLR2/1) and FSL-1 (TLR2/6), which activate pattern recognition receptors associated with gram-positive bacteria, as with LPS, activated whole blood to release PGE_2_, an effect that was abolished by cPLA_2_α deficiency. Neither Poly(I:C), which stimulates the viral pathogen recognition receptor TLR3, nor IL-1β, which works independently of pattern recognition receptors, stimulated PGE_2_ release from whole blood. Although these data demonstrate that cPLA_2_α is central to leukocyte eicosanoid synthesis, particularly for PGE_2_, there are clearly roles for other PLA_2_ isoforms such as sPLA_2_ (13).

To understand the implications of loss of eicosanoid production to the inflammatory response, we measured CXCL8 production, induction of which reflects both primary activation of inflammatory transcriptional pathways such as NF-κB pathways and subsequent secretion of TNF-α and IL-1β (20, 41). Whole blood from healthy volunteers treated with FSL-1, Poly(I:C), or LPS, but not with IL-1β, released the inflammatory chemokine CXCL8. Blood from a cPLA_2_α-deficient patient exhibited more than 5-fold greater responses to all agents except IL-1β as compared with matched controls (Fig. 5*A*). Treatment of blood from healthy volunteers with the COX inhibitor diclofenac suppressed the CXCL8 response to LPS but did not modify CXCL8 release stimulated by other tested agents (Fig. 5*A*), indicating that the effect was not mediated by loss of COX metabolites. Although it cannot be excluded that cPLA_2_α-deficient patient blood contains altered leukocyte subsets, blood constituents, or other confounding influences, these data suggest that cPLA_2_α-dependent mediators, other than COX products, act to suppress cytokine responses by blood leukocytes. This effect may reflect loss of 11- and/or 15-HETE synthesis because these were also detected in LPS-stimulated whole blood, and it has been previously reported that 15-HETE has anti-inflammatory activity (42, 43).

Similarly, endothelial cells from healthy donors released CXCL8 when stimulated with PAMPs directed at TLR2, 3, or 4, or with IL-1β. As with leukocytes in whole blood, endothelial cells from a cPLA_2_α-deficient patient released elevated levels of CXCL8 when stimulated with inflammatory agents, consistent with activation of NF-κB pathways following treatment with inflammatory stimuli, as we have previously described (25). Endothelial cells from either type of donor did not respond to ligands for TLR5, the pattern recognition receptor for motile bacteria and fungi, TLR7 and TLR8, pattern recognition receptors for single-stranded RNA viruses, or TLR9, which is the pattern recognition receptor for bacterial DNA. Importantly, in contrast to blood leukocyte studies, these endothelial cells constitute a single, defined cell type in a controlled medium suggesting that any differences observed likely reflect changes in eicosanoid production as compared to confounding factors present in blood cells. Because prostacyclin was the most abundant eicosanoid produced by endothelial cells and is a powerful inhibitor of vascular inflammation, this proinflammatory phenotype of cPLA_2_α-deficient endothelial cells is most easily explained by loss of this fundamental vascular hormone. CXCL8 is a potent neutrophil chemotactic factor, which has been implicated in atherogenesis (44); thus, augmented production of CXCL8 and potentially other NF-κB–driven cytokines is likely to be detrimental to cardiovascular health. Moreover, taken together, these studies demonstrate that on a global level, blood leukocytes and endothelial cells require cPLA_2_α to produce eicosanoids in response to a range of inflammatory stimuli, and this exerts both COX-dependent and possibly COX-independent regulation of cytokine production and, by inference, immunologic/inflammatory defenses, consistent with clinical manifestations in these patients (18, 19).

### Production *in vivo*

Finally, to provide an overview of the contribution of cPLA_2_α to eicosanoid formation from all sources in the body, we measured the eicosanoid profile in plasma and specific urinary eicosanoid metabolites. Plasma from healthy volunteers contained low levels of primarily metabolites of linoleic acid, EPA, and DHA. Patients lacking cPLA_2_α had reduced levels of these mediators compared with plasma from healthy volunteers. Because cPLA_2_α has strong specificity for arachidonate-containing phospholipids, this may reflect altered physiology in these patients. Notably, basal plasma also contained substantial levels of 12-HETE, which may reflect platelet activation during blood sampling; as noted above, 12-HETE is the major product of activated platelets. However, surprisingly, a small 12-HETE peak was also seen in cPLA_2_α-deficient patient plasma, suggesting possible cPLA_2_α-independent eicosanoid formation in the body.

Interpretation of plasma eicosanoid data as representative of a circulating pool is difficult because levels may reflect local vascular activation during blood sampling, and many eicosanoids rapidly degrade/clear from the circulation. For this reason, many favor measurement of urinary metabolites to assess *in vivo* eicosanoid production. Using this approach, we observed that absence of cPLA_2_α was associated with strong reductions in the levels of LTE_4_, prostacyclin, and TXA_2_ metabolites, consistent with the reductions in TXA_2_ production by platelets, prostacyclin production by endothelial cells, and 5-HETE production by monocytes/neutrophils [LTE_4_ is a downstream metabolite of 5-LOX products (40)] that we noted in our *in vitro* cell studies. Of particular relevance to platelet function was the urinary TXA_2_ metabolite, TX-M. This has been often recommended as a marker of platelet activation *in vivo* that could be used to gauge the efficacy of aspirin treatment and the level of ongoing platelet activation (45). We noted that whereas platelets from cPLA_2_α-deficient patients did not produce TXA_2_, urinary levels of TX-M in the patients were reduced only by ∼50%. This demonstrates that urinary TX-M does not specifically report production from platelets and adds to a growing body of evidence questioning the origin of TX-M and other urinary prostanoid metabolites (23, 46). In addition, substantial levels of PGD_2_, PGE_2_, and 8-isoprostane metabolites remained in urine samples from cPLA_2_α-deficient patients, further suggesting that there are sites in the body where considerable cPLA_2_α-independent prostanoid formation occurs.

## CONCLUSIONS

Here, we have examined the contribution of cPLA_2_α to eicosanoid formation, and thrombotic and inflammatory responses in platelets, blood leukocytes, and endothelial cells from individuals with a unique genetic inactivation of this enzyme. Our data demonstrate an absolute requirement for cPLA_2_α in eicosanoid synthesis in the vascular compartment with a consequent loss of platelet activation pathways, reduced antithrombotic prostacyclin, and increased inflammatory sensitivity of both endothelial cells and leukocytes. This study unites many conflicting observations in the literature and provides a definitive account of the rate-limiting and perhaps most fundamental component of this system, cPLA_2_α.

## Supplementary Material

Supplemental Data

## Abbreviations

CD31: platelet endothelial cell adhesion molecule-1
COX: cyclooxygenase
cPLA_2_: cytosolic phospholipase A_2_
CXCL8: (C-X-C motif) ligand-8
DHA: docosahexaenoic acid
EET: epoxyeicosatrienoic acid
EPA: eicosapentaenoic acid
FLA: flagellin
FSL-1: bisacylated lipoprotein CGDPKHPKSF
HETE: hydroxyeicosatetraenoic acid
iPLA_2_: calcium-independent phospholipase A_2_
LOX: lipoxygenase
LTE_4_: leukotriene E4
Pam3CSK4: triacylated lipoprotein CSK4
PAMP: pathogen-associated molecular pattern
PGD_2_: prostaglandin D_2_
PGE_2_: prostaglandin E_2_
PLA_2_: phospholipase A_2_
Poly(I:C): polyinosinic:polycytidylic acid
prostacyclin: prostaglandin I_2_
sPLA_2_: secreted phospholipase A_2_
TRAP: thrombin receptor-activating peptide
TXA_2_: thromboxane A_2_
TX-M: metabolite of thromboxane A_2_
