# Supplementary material for: Inherited human group IVA cytosolic phospholipase A2 deficiency abolishes platelet, endothelial, and leucocyte eicosanoid generation
Source: FASEB J. 2016 Oct 17;29(11):4568–78. doi: 10.1096/fj.15-275065 (PMC4608906; doi:10.1096/fj.15-275065)
Supplement: Supplemental Data [file supp_29_11_4568__index.html]

Inherited human group IVA cytosolic phospholipase A2 deficiency abolishes platelet, endothelial, and leucocyte eicosanoid generation — Inherited human group IVA cytosolic phospholipase A2 deficiency abolishes platelet, endothelial, and leucocyte eicosanoid generation — Supplemental Data 

# Inherited human group IVA cytosolic phospholipase A2 deficiency abolishes platelet, endothelial, and leucocyte eicosanoid generation

## Supplementary Data

- Supplemental Data
- Supplemental Data
- Supplemental Data
- Supplemental Data

- Facebook
- Google+
- LinkedIn
- Mendeley
- Reddit
- StumbleUpon
- Technorati
- Twitter

What's this?
