# Supplementary material for: Inherited human group IVA cytosolic phospholipase A2 deficiency abolishes platelet, endothelial, and leucocyte eicosanoid generation
Source: FASEB J. 2016 Oct 17;29(11):4568–78. doi: 10.1096/fj.15-275065 (PMC4608906; doi:10.1096/fj.15-275065)
Supplement: Supplemental Data [file supp_fj.15-275065_Supplemental_Figures.pdf]

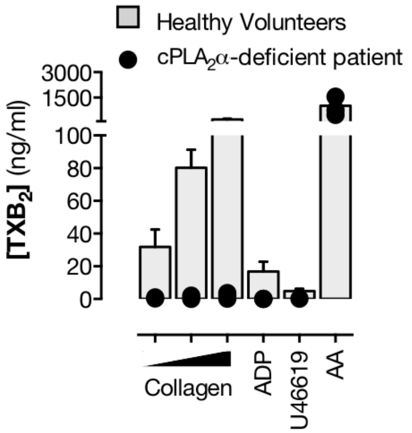

Figure S1. Effect of $cPLA_2$ deficiency on platelet TXA_2 release to a range of agonists. TXA_2 release (measured as TXB_2) by platelet-rich plasma from healthy volunteer or $cPLA_2α$-deficient patients (patient B + patient S) blood in response to stimulation by collagen (0.3-3 µg/ml), ADP (5 µmol/L), U46619 (10 µmol/L) or arachidonic acid (1mmol/L). n=4 (healthy volunteers), n=2 (patient).

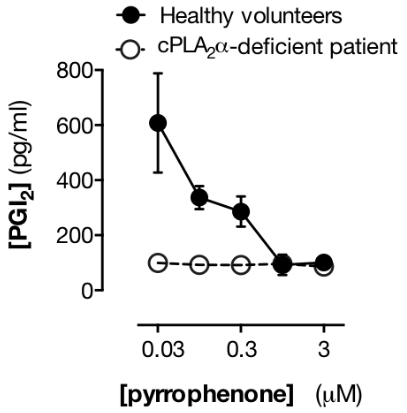

Figure S2. Effect of $cPLA_2$ inhibition on prostacyclin release by endothelial cells. Prostacyclin release (measured as 6-keto-$PGF_{1α}$) by endothelial cells isolation from healthy volunteer or $cPLA_2α$ -deficient patient (patient S) blood in response to A23187 stimulation in the presence of increasing concentrations of the $cPLA_2$ inhibitor, pyrrophenone. n=4 (healthy volunteers), n=1 (patient).
