# Supplementary material for: Inherited human group IVA cytosolic phospholipase A2 deficiency abolishes platelet, endothelial, and leucocyte eicosanoid generation
Source: FASEB J. 2016 Oct 17;29(11):4568–78. doi: 10.1096/fj.15-275065 (PMC4608906; doi:10.1096/fj.15-275065)
Supplement: Supplemental Data [file supp_fj.15-275065_Supplemental_Table1.pdf]

	Vehicle (PBS)		Collagen (30µg/ml)		TRAP6 (30µM)		A23187 (50µM)	
**Mediator (ng/ml)**	**Control**	**$cPLA_2α$ deficient**	**Control**	**$cPLA_2α$ deficient**	**Control**	**$cPLA_2α$ deficient**	**Control**	**$cPLA_2α$ deficient**
6ketoPGF$_{1α}$	0.0 ± 0.0	0.0	0.1 ± 0.0	0.1	0.1 ± 0.0	0.0	0.2 ± 0.1	0.0
TXB_2	0.1 ± 0.0	0.0	17.6 ± 3.1	0.1	24.1 ± 7.5	0.0	38.4 ± 4.4	0.1
PGE_2	0.0 ± 0.0	0.0	1.1 ± 0.2	0.0	2.0 ± 1.0	0.0	4.8 ± 1.2	0.0
PGD_2	0.0 ± 0.0	0.0	0.8 ± 0.2	0.0	1.1 ± 0.5	0.0	2.7 ± 0.6	0.0
8isoPGF$_{2α}$	0.0 ± 0.0	0.0	0.1 ± 0.0	0.0	0.1 ± 0.0	0.0	0.1 ± 0.0	0.0
9,12,13-THOME	1.2 ± 0.9	0.2	0.3 ± 0.1	0.2	1.2 ± 1.0	0.2	1.2 ± 0.9	0.2
9,10,13-THOME	0.1 ± 0.0	0.1	0.1 ± 0.0	0.1	0.1 ± 0.0	0.0	0.2 ± 0.0	0.0
12,13-DHOME	13.8 ± 4.6	2.5	15.0 ± 5.7	3.0	15.8 ± 5.5	1.9	17.3 ± 7.0	1.8
9,10-DHOME	7.6 ± 2.4	2.4	8.1 ± 2.7	2.7	8.8 ± 3.1	1.7	9.8 ± 3.2	1.2
19,20-DiHDPA	0.9 ± 0.2	1.2	0.9 ± 0.2	1.3	1.0 ± 0.2	0.9	0.9 ± 0.2	0.8
14,15-DHET	0.5 ± 0.0	0.3	0.5 ± 0.0	0.3	0.5 ± 0.0	0.2	0.5 ± 0.0	0.2
11,12-DHET	0.3 ± 0.0	0.1	0.3 ± 0.0	0.1	0.3 ± 0.0	0.1	0.3 ± 0.0	0.1
8,9-DHET	0.1 ± 0.0	0.1	0.1 ± 0.0	0.1	0.2 ± 0.0	0.1	0.2 ± 0.0	0.1
5,6-DHET	0.1 ± 0.0	0.1	0.1 ± 0.0	0.1	0.1 ± 0.0	0.0	0.2 ± 0.1	0.0
13-HODE	1.8 ± 0.3	0.6	2.0 ± 0.3	0.7	2.0 ± 0.3	0.6	3.4 ± 0.6	0.6
9-HODE	0.8 ± 0.1	0.2	1.0 ± 0.1	0.2	1.1 ± 0.2	0.1	1.5 ± 0.1	0.1
20-HETE	0.3 ± 0.1	0.1	0.3 ± 0.1	0.0	0.3 ± 0.1	0.1	0.4 ± 0.1	0.0
19-HETE	0.3 ± 0.0	0.2	0.3 ± 0.0	0.2	0.3 ± 0.0	0.1	0.3 ± 0.0	0.1
15-HETE	0.7 ± 0.1	0.5	10.5 ± 2.2	0.6	14.2 ± 4.9	0.4	34.5 ± 2.5	0.7
12-HETE	8 ± 5	2.8	235 ± 64	6.0	43 ± 19	2.9	838 ± 90	25.7
11-HETE	0.1 ± 0.0	0.1	4.1 ± 0.8	0.1	6.2 ± 2.4	0.0	11.3 ± 1.3	0.1
5-HETE	0.7 ± 0.2	0.4	1.3 ± 0.3	0.5	1.2 ± 0.2	0.3	217 ± 39	0.3
12,13-EpOME	2.9 ± 0.6	0.8	2.6 ± 0.4	0.8	2.8 ± 0.5	0.8	2.8 ± 0.5	1.0
9,10-EpOME	0.3 ± 0.1	0.2	0.3 ± 0.0	0.2	0.3 ± 0.1	0.2	0.4 ± 0.1	0.3
19,20-EpDPE	0.4 ± 0.1	0.8	0.5 ± 0.1	1.0	0.5 ± 0.1	0.8	0.6 ± 0.1	0.7
17,18-EpETE	0.0 ± 0.0	0.0	0.0 ± 0.0	0.0	0.0 ± 0.0	0.0	0.0 ± 0.0	0.0
14,15-EET	0.1 ± 0.0	0.0	0.1 ± 0.0	0.1	0.1 ± 0.0	0.0	0.4 ± 0.0	0.1
11,12-EET	0.0 ± 0.0	0.0	0.0 ± 0.0	0.0	0.0 ± 0.0	0.0	0.2 ± 0.0	0.0
8,9-EET	0.1 ± 0.0	0.0	0.1 ± 0.0	0.1	0.1 ± 0.0	0.0	0.2 ± 0.0	0.0
5,6-EET	0.4 ± 0.1	0.2	0.6 ± 0.1	0.3	0.7 ± 0.1	0.2	2.4 ± 0.1	0.4

Table S1. Contribution of $cPLA_2α$ to eicosanoid synthesis in whole blood stimulated with platelet activators. Total eicosanoid levels in whole blood from healthy volunteers ('control') or from patient S lacking $cPLA_2α$ ('$cPLA_2α$ deficient') stimulated with vehicle (PBS), collagen (30µg/ml), TRAP-6 amide (30µM) or Ca^{2+} ionophore A23187 (30µM). n=4 (healthy volunteers), n=1 (patient).
