# Supplementary material for: Inherited human group IVA cytosolic phospholipase A2 deficiency abolishes platelet, endothelial, and leucocyte eicosanoid generation
Source: FASEB J. 2016 Oct 17;29(11):4568–78. doi: 10.1096/fj.15-275065 (PMC4608906; doi:10.1096/fj.15-275065)
Supplement: Supplemental Data [file supp_fj.15-275065_Supplemental_Table2.pdf]

	Vehicle (PBS)		AA (1mM)	
Mediator (ng/ml)	Control	cPLA$_2$α deficient	Control	cPLA$_2$α deficient
6ketoPGF$_{1\alpha}$	0.0 ± 0.0	0.0	2.5 ± 0.6	0.5
TXB$_2$	0.1 ± 0.0	0.0	168.0 ± 12.1	83.3
PGE$_2$	0.0 ± 0.0	0.0	144.7 ± 38.8	30.1
PGD$_2$	0.0 ± 0.0	0.0	143.1 ± 46.5	35.0
8isoPGF$_{2\alpha}$	0.0 ± 0.0	0.0	14.3 ± 5.9	0.8
9,12,13-THOME	1.2 ± 0.9	0.2	1.4 ± 0.5	0.2
9,10,13-THOME	0.1 ± 0.0	0.1	0.9 ± 0.4	0.1
12,13-DHOME	13.8 ± 4.6	2.5	18.0 ± 2.9	2.4
9,10-DHOME	7.6 ± 2.4	2.4	17.2 ± 7.2	2.2
19,20-DiHDPA	0.9 ± 0.2	1.2	1.0 ± 0.1	1.1
14,15-DHET	0.5 ± 0.0	0.3	23.8 ± 6.0	6.7
11,12-DHET	0.3 ± 0.0	0.1	14.1 ± 2.9	6.4
8,9-DHET	0.1 ± 0.0	0.1	8.0 ± 2.7	2.1
5,6-DHET	0.1 ± 0.0	0.1	41.9 ± 16.7	11.6
13-HODE	1.8 ± 0.3	0.6	13.3 ± 5.3	0.7
9-HODE	0.8 ± 0.1	0.2	12.4 ± 5.6	0.4
20-HETE	0.3 ± 0.1	0.1	6.3 ± 0.4	5.1
19-HETE	0.3 ± 0.0	0.2	9.6 ± 3.8	1.7
15-HETE	0.7 ± 0.1	0.5	1945.3 ± 484.6	813.3
12-HETE	8.5 ± 5.1	2.8	1448.3 ± 228.7	1060.0
11-HETE	0.1 ± 0.0	0.1	301.1 ± 83.3	129.3
5-HETE	0.7 ± 0.2	0.4	520.0 ± 148.3	298.7
12,13-EpOME	2.9 ± 0.6	0.8	4.1 ± 0.8	0.7
9,10-EpOME	0.3 ± 0.1	0.2	1.2 ± 0.5	0.2
19,20-EpDPE	0.4 ± 0.1	0.8	1.1 ± 0.5	1.0
17,18-EpETE	0.0 ± 0.0	0.0	0.2 ± 0.1	0.1
14,15-EET	0.1 ± 0.0	0.0	149.8 ± 36.8	66.3
11,12-EET	0.0 ± 0.0	0.0	113.5 ± 34.9	38.7
8,9-EET	0.1 ± 0.0	0.0	115.9 ± 42.2	40.9
5,6-EET	0.4 ± 0.1	0.2	471.8 ± 201.7	200.3

Table S2. Contribution of cPLA$_2$α to eicosanoid synthesis in whole blood stimulated with exogenous arachidonic acid (AA). Total eicosanoid levels in whole blood from healthy volunteers ('control') or from patient S, lacking cPLA$_2$α ('cPLA$_2$α deficient') stimulated with vehicle (PBS) or exogenous AA (1mM). n=4 (healthy volunteers), n=1 (patient).
