# Supplementary material for: Inherited human group IVA cytosolic phospholipase A2 deficiency abolishes platelet, endothelial, and leucocyte eicosanoid generation
Source: FASEB J. 2016 Oct 17;29(11):4568–78. doi: 10.1096/fj.15-275065 (PMC4608906; doi:10.1096/fj.15-275065)
Supplement: Supplemental Data [file supp_fj.15-275065_Supplemental_Table3.pdf]

	Vehicle (DMEM)		LPS (10μg/ml)		LPS + AA (1mM)	
**Mediator (ng/ml)**	**Control**	**cPLA$_2$α deficient**	**Control**	**cPLA$_2$α deficient**	**Control**	**cPLA$_2$α deficient**
6ketoPGF$_{1α}$	0.0 ± 0.0	0.0	0.0 ± 0.0	0.0	0.3 ± 0.0	0.1
TXB$_2$	0.6 ± 0.3	0.0	2.5 ± 0.4	0.3	128.1 ± 12.9	35.2
PGE$_2$	5.7 ± 5.3	0.1	26.2 ± 6.7	2.0	161.6 ± 25.3	55.4
PGD$_2$	0.1 ± 0.0	0.0	0.2 ± 0.1	0.1	140.9 ± 5.2	105.1
8isoPGF$_{2α}$	0.0 ± 0.0	0.0	0.2 ± 0.0	0.0	2.7 ± 0.2	1.4
9,12,13-THOME	0.7 ± 0.5	0.2	0.7 ± 0.4	0.3	0.7 ± 0.4	0.3
9,10,13-THOME	0.0 ± 0.0	0.0	0.0 ± 0.0	0.0	0.1 ± 0.0	0.1
12,13-DHOME	21.8 ± 5.6	3.0	19.6 ± 3.8	3.9	12.0 ± 6.3	3.6
9,10-DHOME	9.6 ± 3.2	2.5	8.5 ± 3.0	3.6	5.1 ± 3.0	0.9
19,20-DiHDPA	2.2 ± 0.5	2.9	2.1 ± 0.2	2.8	1.4 ± 0.6	1.9
14,15-DHET	0.7 ± 0.1	0.6	0.8 ± 0.1	0.5	8.9 ± 6.2	5.7
11,12-DHET	1.1 ± 0.1	0.5	0.8 ± 0.1	0.7	7.7 ± 5.2	6.2
8,9-DHET	1.2 ± 0.1	0.9	1.3 ± 0.1	0.6	7.4 ± 3.3	5.1
5,6-DHET	1.3 ± 0.2	0.5	1.3 ± 0.1	0.8	50.1 ± 23.9	23.9
13-HODE	16.8 ± 3.8	7.3	30.9 ± 9.7	20.7	12.8 ± 4.3	19.0
9-HODE	4.9 ± 1.1	1.6	5.3 ± 1.5	4.1	3.8 ± 1.6	0.5
20-HETE	0.8 ± 0.4	0.0	0.6 ± 0.2	0.0	9.9 ± 3.9	9.6
19-HETE	0.5 ± 0.3	0.0	0.8 ± 0.4	1.2	18.1 ± 6.3	18.8
15-HETE	11.9 ± 4.7	5.9	34.8 ± 8.6	12.5	2787 ± 1116	3386.7
12-HETE	215.0 ± 50.2	71.5	229.2 ± 70.6	98.1	1758 ± 1029	2082.0
11-HETE	3.6 ± 2.1	0.7	8.8 ± 4.2	2.5	400 ± 213.9	595.3
5-HETE	4.5 ± 2.0	2.3	4.8 ± 1.0	2.4	797.1 ± 359.8	1045.3
12,13-EpOME	2.8 ± 0.5	1.7	2.7 ± 0.7	1.9	1.8 ± 0.5	1.8
9,10-EpOME	0.6 ± 0.1	0.5	0.6 ± 0.2	0.6	0.4 ± 0.1	0.6
19,20-EpDPE	1.3 ± 0.2	4.4	1.6 ± 0.2	4.8	1.4 ± 0.5	5.2
17,18-EpETE	0.0 ± 0.0	0.0	0.0 ± 0.0	0.0	0.0 ± 0.0	0.0
14,15-EET	0.0 ± 0.0	0.0	0.0 ± 0.0	0.0	63.3 ± 18.1	61.0
11,12-EET	0.0 ± 0.0	0.0	0.0 ± 0.0	0.0	25.8 ± 9.1	33.9
8,9-EET	0.0 ± 0.0	0.0	0.0 ± 0.0	0.0	32.2 ± 11.6	40.3
5,6-EET	0.3 ± 0.3	0.0	0.5 ± 0.3	0.0	119.1 ± 36.6	125.8

Table S3. Contribution of cPLA$_2$α to eicosanoid synthesis in whole blood stimulated with LPS. Total eicosanoid levels in whole blood from healthy volunteers ('control') or from patient S lacking cPLA$_2$α ('cPLA$_2$α deficient') stimulated for 18 hours with vehicle (DMEM) or LPS (10μg/ml) with or without addition of exogenous arachidonic acid (AA; 1mM) for the final 30 mins. n=4 (healthy volunteers), n=1 (patient).
